# A Concise Review on the Physicochemical Properties of Biopolymer Blends Prepared in Ionic Liquids

**DOI:** 10.3390/molecules26010216

**Published:** 2021-01-04

**Authors:** Ahmad Adlie Shamsuri, Khalina Abdan, Tatsuo Kaneko

**Affiliations:** 1 Laboratory of Biocomposite Technology, Institute of Tropical Forestry and Forest Products, Universiti Putra Malaysia, UPM Serdang 43400, Selangor, Malaysia; 2 Energy and Environment Area, Japan Advanced Institute of Science and Technology, 1-1 Asahidai, Nomi District 923-1292, Ishikawa, Japan; kaneko@jaist.ac.jp

**Keywords:** imidazolium, ionic liquid, biopolymer, blend, physicochemical

## Abstract

An enhancement of environmental concern lately has improved the awareness of researchers in employing eco-friendly solvents for processing biopolymers. Recently, ionic liquids have been utilized to prepare biopolymer blends as they are non-volatile and recyclable. Biopolymers such as cellulose, chitin, chitosan, keratin, lignin, silk, starch, and zein are widely used for the preparation of biopolymer blends via dissolution in ionic liquids, followed by coagulation procedure. In this concise review, three types of ionic liquids based on imidazolium cations combined with different counter anions that are frequently utilized to prepare biopolymer blends are described. Moreover, three types of biopolymer blends that are prepared in ionic liquids were classified, specifically polysaccharide/polysaccharide blends, polysaccharide/polypeptide blends, and polysaccharide/bioplastic blends. The physicochemical properties of biopolymer blends prepared in different imidazolium-based ionic liquids are also concisely reviewed. This paper may assist the researchers in the polymer blend area and generate fresh ideas for future research.

## 1. Introduction

**Figure 1:**
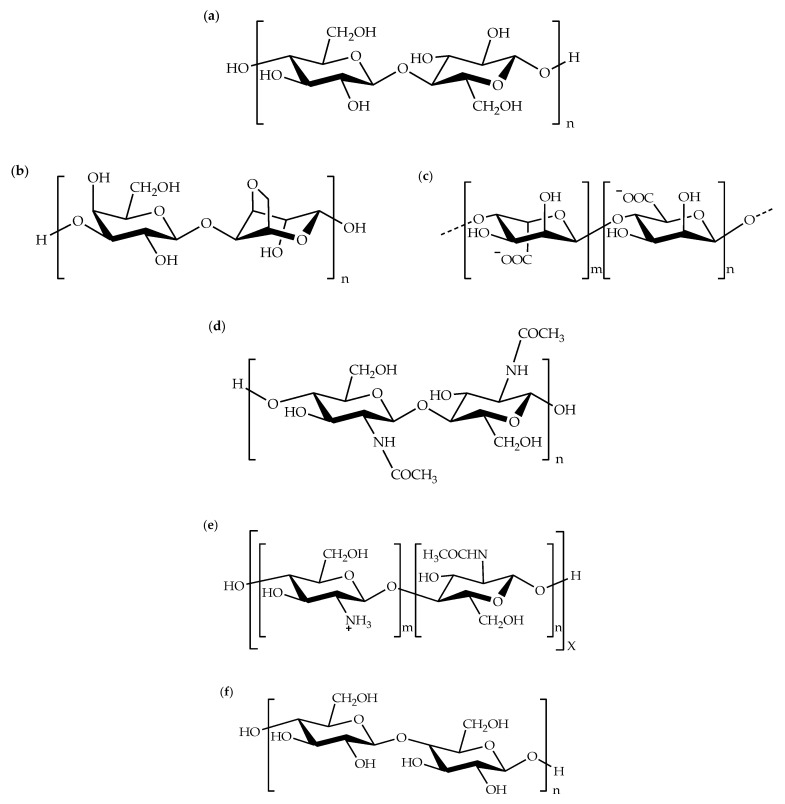
Chemical structures of (**a**) cellulose, (**b**) agarose, (**c**) alginate, (**d**) chitin, (**e**) chitosan, and (**f**) starch.

**Figure 2:**
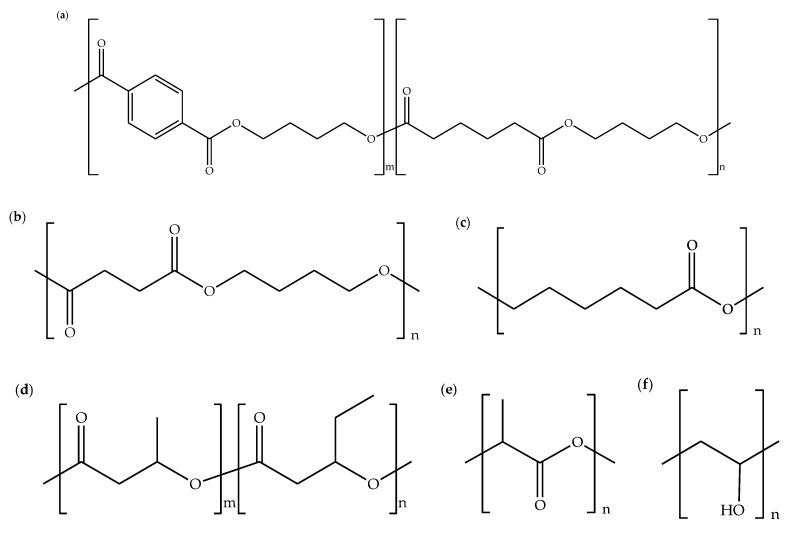
Chemical structures of (**a**) poly(butylene-adipate-co-terephtalate) (PBAT), (**b**) poly(butylene succinate) (PBS), (**c**) polycaprolactone (PCL), (**d**) poly(3-hydroxybutyrate-co-3-hydroxyvalerate) (PHBV), (**e**) polylactide (PLA), and (**f**) poly(vinyl alcohol) (PVA).

Nowadays, the utilization of biopolymers in the packaging industry has turned out to be a responsibility of the manufacturer due to the environmental awareness and rising of the biodegradable polymers industry. Furthermore, the sustainability of biopolymers supplies is also attributed to the development of the industry. Biopolymers are polymeric natural materials acquired from bio-based resources. They become the preferred choice for polymer blends due to their biodegradability, renewability, economy, and availability. Cellulose was mainly exploited for the preparation of biopolymer blends in ionic liquids. Cellulose in the form of pulp [1,2,3,4,5,6,7,8], linter [9,10], microgranular [11,12,13], microcrystalline [14,15,16,17,18,19,20,21,22,23,24,25,26,27,28], and fabric [29] were usually used as raw materials for preparing biopolymer blends. On top of that, other biopolymers such as agarose [30], alginate [31,32], chitin [10,17,26,27,31,33], chitosan [1,4,5,7,29,30,34,35,36], chondroitin sulfate [37], collagen [24,32], keratin [6,8,21], lignin [3,20,38], silk [2,14,15,18,19,22,25,28,35], starch [16,39,40,41,42], xylan [20,43], xyloglucan [23], and zein [16,39,41] were employed as blend components. Figure 1 shows the chemical structures of some biopolymers. On the other hand, synthetic biodegradable polymers (bioplastics), for example, poly(butylene-adipate-co-terephtalate) (PBAT) [38,44,45], poly(butylene succinate) (PBS) [40,46], polycaprolactone (PCL) [11,47], poly(3-hydroxybutyrate-co-3-hydroxyvalerate) (PHBV) [13], polylactide (PLA) [33,38,44,45,47], and poly(vinyl alcohol) (PVA) [12] were also used as components for biopolymer blends. Figure 2 displays the chemical structures of PBAT, PBS, PCL, PHBV, PLA, and PVA.

**Figure 3:**
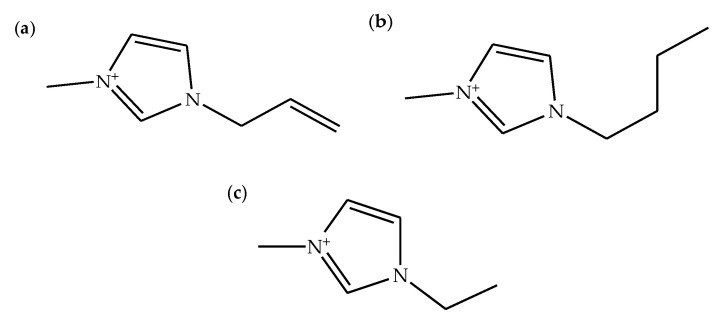
Chemical structures of (**a**) 1-allyl-3-methylimidazolium cation, (**b**) 1-butyl-3-methylimidazolium cation, and (**c**) 1-ethyl-3-methylimidazolium cation.

Ionic liquids are ionic compounds that usually have a low melting point (<100 °C). They possess bigger cations and/or anions than common ionic compounds. Ionic liquids are also non-volatile compounds as they have a lower vapor pressure compared to volatile organic compounds [48]. They are typically regarded as environmentally friendly solvents because of their recyclability. Ionic liquids could be recycled by removing unwanted solvent through evaporation process [12]. Additionally, ionic liquids have extraordinary solvent properties, for example, good miscibility with many organic solvents, high thermal stability, good electrical conductivity, high polarity, and non-flammability. They also have the ability to dissolve most biopolymers and organic materials, as well as some inorganic materials. Moreover, ionic liquids could be customized according to the needs of the uses [49]. Among many types of ionic liquids, imidazolium-based ionic liquids with different counter anions are frequently utilized for dissolution of biopolymers. Ionic liquids with methylimidazolium cation such as 1-allyl-3-methylimidazolium [19,29], 1-butyl-3-methylimidazolium [9,30], and 1-ethyl-3-methylimidazolium [5,7] (Figure 3) have effectively dissolved different types of biopolymers.

**Figure 4:**
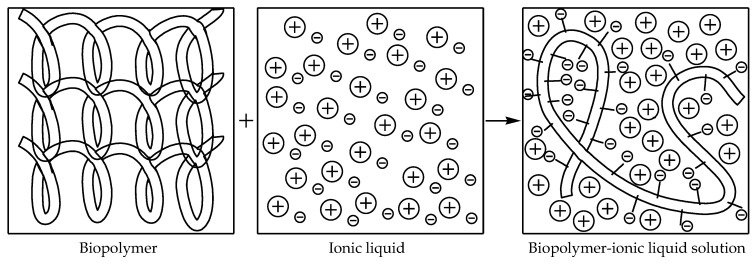
Mechanism of the dissolution process of the biopolymer in ionic liquid.

In the last decade, several dissolution approaches have been studied with the purpose of combining the different biopolymers for producing biopolymer blends. The utilization of organic compounds such as *N,N*-dimethylacetamide/LiCl, *N*-methylmorpholine *N*-oxide, and cuoxam for dissolution of biopolymers have been identified in the preceding research [14]. However, there are some disadvantages that were discovered, for example, non-eco-friendly, toxic, expensive, etc. Furthermore, the significant reduction of the molecular weight of biopolymers has also commonly been found by using such rough compounds [14]. The eco-friendly dissolution approach, by using ionic liquids, is a practical method to prepare biopolymer blends because of their non-volatility and recyclability. Figure 4 demonstrates the mechanism of the dissolution process of the biopolymer in ionic liquid. Hitherto, to the best of the authors’ knowledge, a concise review on the physicochemical properties (e.g., mechanical, thermal, crystalline, chemical, etc. [50]) of biopolymer blends prepared in ionic liquids has not yet been reported. That is the reason of composing a systematized review in this paper. The selection of biopolymer blends in this concise review is not only based on biopolymer blends prepared in imidazolium-based ionic liquids but also based on the studied physicochemical properties.

## 2. Types of Imidazolium-Based Ionic Liquids in Dissolution of Biopolymers

### 2.1. 1-Allyl-3-Methylimidazolium-Based Ionic Liquids

**Figure 5:**
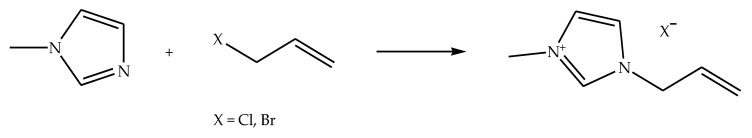
Schematic of the alkylation of *N*-methylimidazole with allyl halide to prepare 1-allyl-3-methylimidazolium halide.

1-Allyl-3-methylimidazolium-based ionic liquids can be synthesized through alkylation reaction by reacting *N*-methylimidazole with alkylating agents such as allyl halides. Figure 5 shows the schematic of the alkylation of *N*-methylimidazole with allyl halide to prepare 1-allyl-3-methylimidazolium halide. The alkylation of *N*-methylimidazole is usually conducted under reflux condition at elevated temperature with stirring [1]. Table 1 demonstrates the 1-allyl-3-methylimidazolium-based ionic liquids with different counter anions utilized for dissolution of biopolymers. It can be seen that the [Amim][Cl] has typically been utilized for biopolymers dissolution compared to [Amim][Br]. This is possibly due to most of the biopolymers have a better affinity with [Amim][Cl] than that of [Amim][Br]. Nonetheless, the solubility of biopolymers in [Amim][Cl] depends on their molecular mass, whereby the lower molecular mass biopolymers are easy to dissolve, and vice versa [51]. On the other hand, cellulose is commonly dissolved in [Amim][Cl] in comparison with other biopolymers.

Cellulose has a greater tendency to be dissolved in [Amim][Cl] compared to chitin and chitosan. One of the reasons is the van der Waals energy contribution between the glucose and imidazolium cations [19]. According to a review article written by other researchers, they have found that the cellulose can be dissolved in [Amim][Cl] up to 14.5 wt.% at 130 °C [52]. In addition, cellulose dissolved in [Amim][Cl] had no derivatization as confirmed by Fourier transform infrared (FTIR) spectroscopy [29] and maintain its final molecular weight [18,25]. Dissolution of cellulose in [Amim][Cl] occurs due to the presence of interactions between the hydroxyl groups of cellulose and anions, as well as cations of [Amim][Cl], which causes the breakage of the hydrogen bonding between the molecular chains of cellulose [29]. Moreover, in Table 1, it can be observed that the [Amim][Cl] also regularly utilized for dissolution of silk. This is due to the presence of strong interactions between the allyl group and silk molecule [22]. Additionally, [Amim][Cl] could dissolve keratin such as hair fibers up to 19 wt.% at 130 °C [6]. The solutions of [Amim][Cl]-biopolymers could be employed for the production of gels, coatings, and films [53].

### 2.2. 1-Butyl-3-Methylimidazolium-Based Ionic Liquids

1-Butyl-3-methylimidazolium chloride can also be synthesized via alkylation reaction by reacting *N*-methylimidazole with butyl chloride. Table 2 displays the 1-butyl-3-methylimidazolium-based ionic liquids with different counter anions utilized for dissolution of biopolymers. It can be perceived that the [Bmim][Cl] is frequently utilized for dissolution of cellulose in comparison with other biopolymers. This is because of the high chloride content in [Bmim][Cl], which could efficiently break down the hydrogen bonding network of cellulose [13]. The previous study exhibited that the cellulose pulp could be dissolved in [Bmim][Cl] up to 25 wt.% at 90 °C [11]. Moreover, [Bmim][Cl] is able to dissolve chitosan up to 10 wt.% at 110 °C for 5 h with stirring [5]. In addition, the keratin, for example, hair fibers could be dissolved in [Bmim][Cl] up to 13 wt.% at 130 °C [6]. Furthermore, like biopolymers, bioplastics such as PCL, PVA, and PHBV could also be dissolved in [Bmim][Cl] [11,12,13]. On top of that, the use of [Bmim][Cl] not only limited as a solvent to dissolve biopolymers for preparation of biopolymer blends, but it can also be used as a compatibilizer for corn starch/corn zein incompatible blends [39,41,54], and as a plasticizer for PBS/starch blends [40].

**Figure 6:**
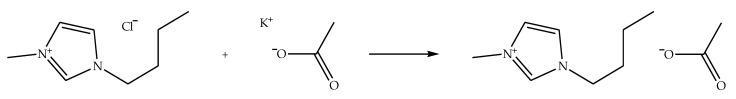
Schematic of the anion exchange of 1-butyl-3-methylimidazolium chloride with potassium acetate to prepare 1-butyl-3-methylimidazolium acetate.

On the other hand, 1-butyl-3-methylimidazolium acetate can be synthesized from 1-butyl-3-methylimidazolium chloride through metathesis reaction [55] by exchanging chloride anion with an acetate anion using potassium acetate. Figure 6 indicates the schematic of the anion exchange of 1-butyl-3-methylimidazolium chloride with potassium acetate to prepare 1-butyl-3-methylimidazolium acetate. The ion exchange is commonly carried out in a polar solvent such as ethanol at room temperature with stirring [56]. In Table 2, it can also be seen that unlike the [Bmim][Cl], [Bmim][OAc] is able to dissolve the wide-range molecular weight of chitosan [4] due to it possessing lower viscosity that facilitates its dissolution ability [52]. The intra- and intermolecular hydrogen bonding interactions existent in the structure of cellulose and chitosan could completely be disrupted by [Bmim][OAc] for giving more efficient dissolution [4] compared to [Bmim][Cl] [35]. Besides, the formation of new strong intermolecular hydrogen bonding between the acetate anion and hydroxyl groups of the chitosan occurs during dissolution [35], which induces more content of chitosan dissolves in [Bmim][OAc] [51].

### 2.3. 1-Ethyl-3-Methylimidazolium-Based Ionic Liquids

1-Ethyl-3-methylimidazolium chloride can be synthesized through alkylation reaction as well by reacting *N*-methylimidazole with 1-chloroethane in acetonitrile [57]. Table 3 shows the 1-ethyl-3-methylimidazolium-based ionic liquids with different counter anions utilized for dissolution of biopolymers. It can be observed that the [Emim][Cl] could dissolve cellulose and silk, it capable of dissolving silk up to 23.3 wt.% at 100 °C [51]. Although ionic liquids with chloride anion such as [Amim][Cl] and [Bmim][Cl] are frequently utilized in biopolymers dissolution, however [Emim][Cl] is not extensively used for such application as other 1-ethyl-3-methylimidazolium-based ionic liquids. This is probably due to [Emim][Cl] possesses a higher melting point (>75 °C), thus providing biopolymer solutions with high viscosity and limiting their usage. On top of that, 1-ethyl-3-methylimidazolium acetate or propionate not only can be synthesized via metathesis reaction but also through acid-base reaction by neutralizing 1-ethyl-3-methylimidazolium hydroxide with carboxylic acids such as acetic or propionic acids.

**Figure 7:**
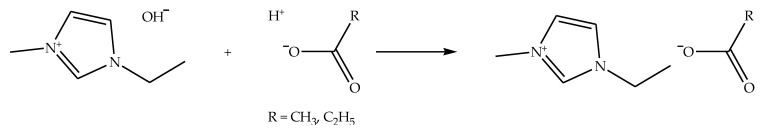
Schematic of the neutralization of 1-ethyl-3-methylimidazolium hydroxide with carboxylic acid to prepare 1-ethyl-3-methylimidazolium carboxylate.

Figure 7 demonstrates the schematic of the neutralization of 1-ethyl-3-methylimidazolium hydroxide with carboxylic acid to prepare 1-ethyl-3-methylimidazolium carboxylate. The reaction is generally performed in a polar solvent like ethanol with stirring at room temperature [56]. In Table 3, it can also be perceived that the [Emim][OAc] is typically utilized for dissolution of many types of biopolymers than [Emim][Cl] and [Emim][OPr]. Additionally, [Emim][OAc] has a lower melting point, lower toxicity, and better biodegradability than ionic liquids consist of halide anions [58]. Moreover, [Emim][OAc] could dissolve cellulose up to 15 wt.% at 90 °C, which is three times than dissolution in [Emim][Cl] at the same temperature [52]. Other previous research showed that the high content of shrimp shell (which contained the high composition of chitin) could considerably be dissolved in [Emim][OAc] up to 46 wt.% at 100 °C in comparison with [Emim][Cl] (9.7 wt.%) and [Bmim][Cl] (10 wt.%) [51]. This is attributed to the greater capability of acetate anion in the dissolution of biopolymers compared to chloride anion [7]. Another carboxylate ionic liquid like [Emim][OPr] could also dissolve cellulose and chitin [10], as it is able to dissolve up to 14 wt.% of chitin at 110 °C [59].

## 3. Types of Biopolymer Blends Prepared in Ionic Liquids

### 3.1. Polysaccharide/Polysaccharide Blends

Blending the polysaccharide with other polysaccharides or polymers is one of the ways for improving their physicochemical properties [15,18]. In this concise review, biopolymer blends have been classified into three types, namely polysaccharide/polysaccharide blends, polysaccharide/polypeptide blends, and polysaccharide/bioplastic blends. The classification is based on the types of blend components. Polysaccharide/polysaccharide blends are the most common biopolymer blends that regularly prepared in ionic liquids. Table 4 displays the polysaccharides, ionic liquids, processing temperatures, and coagulation agents used for preparation of biopolymer blends. It can be seen that two different polysaccharides are usually blended in ionic liquids. Nevertheless, the intractable polysaccharide like cellulose is frequently preferred as the main blend component due to it is easier to dissolve in ionic liquids under mild conditions than that of conventional organic solvent. Additionally, cellulose is the greatest abundant biopolymer in the world, as well as widely used in the paper, polymer, textile, and food industries compared to other biopolymers [60].

On the other hand, the use of ionic liquids as the processing solvent for polysaccharides has produced biodegradable blends in diverse solid shapes, for example, films, fibers, blocks, etc. [12]. Polysaccharide/polysaccharide blends could be prepared by utilizing two different ionic liquids [17]. However, the utilization of sole ionic liquid is a more practical option for preparation of biopolymer blends, hence reducing the complexity of recycling the ionic liquid. In Table 4, it can also be observed that the processing temperature for dissolution and blending of the polysaccharides is relatively high (>84 °C); this indicates that the elevated temperature plays an important role in preparing biopolymer blends. Nonetheless, compared with other polysaccharides, chitosan could degrade at high temperature [5]. Therefore, the maximum temperature for blending chitosan in ionic liquids is 110 °C. On top of that, after the dissolution and blending of the polysaccharides in ionic liquids, a coagulation agent is needed for regenerating the biopolymer blends and removing out the ionic liquid from the blends. In general, deionized or distilled water is favored as the coagulation agent [13] because it is cheap. In addition, methanol, ethanol, or a mixture of alcohol/water can also be used as coagulation agents for such procedure.

### 3.2. Polysaccharide/Polypeptide Blends

Polysaccharides can also be blended with proteins (scientifically known as polypeptides) in ionic liquids to prepare polysaccharide/polypeptide blends [61]. Table 5 indicates the polysaccharides, polypeptides, ionic liquids, processing temperatures, and coagulation agents used for preparation of biopolymer blends. It can be perceived that the polysaccharide like cellulose was also used as a blend component, including chitin and chitosan [19,35]. In contrast, polypeptides such as keratin and silk were usually used as blend components. Ionic liquids, for instance, [Amim][Cl], [Bmim][Cl] and [Emim][OAc] are commonly utilized in preparing polysaccharide/polypeptide blends. Cellulose and keratin could be blended in [Emim][OAc] at 130 °C; this exhibits that the blending of both components requires high temperature for fully dissolved. Besides, any remaining water from cellulose or keratin is able to vaporize at elevated temperature [8]. Instead, the use of [Bmim][Cl] in the preparation of cellulose/keratin blends could be carried out at 120 °C [6]. Therefore, the types of ionic liquids have influenced the processing temperature for blending polysaccharide and polypeptide.

In Table 5, it can also be seen that the polypeptide like silk is broadly used to prepare polysaccharide/polypeptide blends in comparison with keratin. This is possibly attributed to the dissolution of silk requires a slightly lower temperature than that of keratin. For example, cellulose/silk blends could be prepared in [Bmim][Cl] at a temperature of 80 °C [14], which is 40 °C less than the temperature for preparing cellulose/keratin blends (120 °C) in the same ionic liquid [6]. Nevertheless, the use of chitin or chitosan for the preparation of polysaccharide/polypeptide blends could slightly increase the processing temperature. This is because of the solubility of the two polysaccharides in ionic liquids differs from that of cellulose. On the other hand, coagulation agents such as water, methanol, and ethanol can also be employed for regenerating polysaccharide/polypeptide blends, as well as removing ionic liquids since they are completely miscible with coagulation agents at any ratio. During the coagulation procedure, the anions and cations of ionic liquids promptly migrate from the biopolymer blend solution to the coagulation agent; this induces the regeneration of a biopolymer blend gel. After the elimination of the coagulation agent, the formation of the solid biopolymer blend occurs [15].

### 3.3. Polysaccharide/Bioplastic Blends

Polysaccharides can also be blended with synthetic biodegradable polymers (sometimes called bioplastics). Unlike polysaccharide/polysaccharide blends and polysaccharide/polypeptide blends, very few studies have prepared the polysaccharide/bioplastic blends. This is most probably because the bioplastics’ nature is not the same as certain intractable polysaccharides as they can be processed like typical thermoplastics [46]. There are some benefits of blending the polysaccharide with a bioplastic, specifically biodegradability, since both polymers are biodegradable; therefore, their blends can be biodegradable as well [11]. Furthermore, the combination between the polysaccharide and bioplastic could reduce dependence on the expensive bioplastics for the production of environmentally friendly products. This combination is also economical and timesaving compared to the development of low-cost bioplastics. Table 6 demonstrates the polysaccharides, bioplastics, ionic liquids, processing temperatures, and coagulation agents used for preparation of biopolymer blends. It can be noticed that the bioplastics such as PCL, PVA, and PHBV could be blended with cellulose in [Bmim][Cl] at 90–100 °C. This proves that the ionic liquid could effectively dissolve not only natural polymers but also synthetic polymers.

Meanwhile, bioplastic like PLA could be dissolved in [Emim][OAc] and blended with chitin at 100 °C. This indicates that the [Emim][OAc] is a powerful solvent [7] since it can dissolve non-polar hydrophobic polymer as PLA, which is usually soluble in a non-polar solvent like chloroform [33]. The aforementioned intriguing properties of [Emim][OAc] have caused it extensively utilized in the preparation of biopolymer blends. Besides that, there is a formation of intermolecular interactions between the polysaccharides and bioplastics [11,33], and this could affect the physicochemical properties of biopolymer blends. In Table 6, it can also be seen that the coagulation agents such as water and ethanol can be used to regenerate biopolymer blends, as well as the removal of ionic liquids. The ionic liquid can be recycled by evaporating major quantity of coagulation agent under reduced pressure. Moreover, the residual trace of the coagulation agent can be removed by freeze-drying recovered ionic liquid under vacuum pressure [12]. In fact, the molecular structure and dissolution ability of the recycled ionic liquid are the same as the new ionic liquid [12].

## 4. Physicochemical Properties of Biopolymer Blends

### 4.1. Physicochemical Properties of Polysaccharide/Polysaccharide Blends

Table 7 indicates the physicochemical properties of polysaccharide/polysaccharide blends prepared in imidazolium-based ionic liquids. The cellulose/chitin blend fibers have been prepared in the [Emim][OPr] ionic liquid at different biopolymer weight ratios [10]. The mechanical and crystalline properties of the blend fibers were measured and analyzed by means of tensile tester and wide-angle X-ray diffractometer, respectively. The mechanical properties such as Young’s modulus of the cellulose/chitin blend fiber (75:25) increased by up to 24% compared to the pure cellulose fiber. This is due to the higher orientation and crystallinity of the blend fiber than that of the cellulose fiber. On the other hand, the crystalline properties such as crystallinity index of the blend fiber (75:25) moderately increased by up to 6.1% in comparison with the pure cellulose. This is attributed to the fiber spinning and drawing, although no improvement in fiber orientation was perceived for the blend fibers compared to cellulose fibers spun in the same spinning settings [10]. Therefore, it can be deduced that the preparation of cellulose/chitin blend fibers in the [Emim][OPr] ionic liquid has provided biopolymer blends with high modulus and moderate crystallinity properties.

The cellulose/chitosan blend films have been prepared in the [Emim][OAc] ionic liquid at different biopolymer weight ratios [7]. The mechanical, thermal, crystalline, and chemical properties of the blend films were characterized by using testing machine, thermogravimetric analyzer, X-ray diffractometer, and FTIR spectrometer, respectively. The mechanical properties such as tensile strength and elongation at break of the blend films decreased due to the increase of chitosan content, which induced brittleness to the blend films. Nevertheless, the thermal properties such as maximum decomposition temperature of the cellulose/chitosan blend film (1:3) increased by up to 18% compared to the regenerated cellulose. This is caused by the excellent compatibility of the blend solutions, which prompted the creation of special interactions in the blend films. On the other hand, the crystalline properties such as crystallinity pattern of the blend film decreased attributed to the presence of intermolecular interactions between the cellulose and chitosan. Moreover, the chemical properties such as absorption bands of the C=O stretching of the amide I groups of the blend films shifted to higher wavenumber regions in comparison with the C=O stretching band of the regenerated cellulose. On the contrary, absorption bands of the –NH bending of the amide II groups of the blend films shifted to lower wavenumber regions with increasing cellulose content. This indicated that the intermolecular hydrogen bonding interactions have formed between the cellulose and chitosan [7]. Thus, it can be inferred that the preparation of cellulose/chitosan blend films in the [Emim][OAc] ionic liquid has granted well-mixed biopolymer blends with high thermal decomposition and good compatibility.

Meanwhile, the cellulose/chitosan blend sponges have been prepared in the [Amim][Cl] ionic liquid at different biopolymer weight percentages [29]. The mechanical and chemical properties of the blend sponges were measured and analyzed by means of universal testing machine and FTIR spectrometer, respectively. The mechanical properties such as tensile strength at break of the cellulose/chitosan blend sponge (3:1) increased by up to 256% compared to the regenerated cellulose. This is due to the good interface between the cellulose and chitosan, which resulted in high breaking strength of the blend sponge. On the other hand, the chemical properties such as absorption band of the –OH stretching of the hydroxyl group of the blend sponge (3:1) shifted to a higher wavenumber region in comparison with the –OH stretching band of the regenerated cellulose. Additionally, the absorption band of the –NH bending of the amide II group of the blend shifted to a higher wavenumber region compared to the –NH bending band of the chitosan powders. This proved that the formation of intermolecular hydrogen bonding interactions between the –OH groups of cellulose and the –OH and –NH groups of chitosan [29]. Hence, it can be concluded that the preparation of cellulose/chitosan blend sponges in the [Amim][Cl] ionic liquid has given biopolymer blends with high tensile strength and effective interactions.

On top of that, the cellulose/chitosan blend films have been prepared in the [Bmim][OAc] ionic liquid at different biopolymer weight ratios [4]. The thermal, crystalline, and chemical properties of the blend films were characterized by using thermogravimetric analyzer, X-ray diffractometer, and FTIR spectrometer, respectively. The thermal properties such as thermal decomposition of the cellulose/chitosan blend film (90:10) moderately increased by up to 6.9% compared to the cellulose film. This is caused by the presence of chitosan has slowed down the thermal decomposition of the blend film and increased its thermal stability. This is also affected by complementary interactions between the cellulose and chitosan. Nevertheless, the crystalline properties such as crystalline reflection peaks of the blend films decreased due to the suppression of crystallization and establishment of the amorphous phase. On the other hand, the chemical properties such as absorption bands of the C=O stretching of the amide I groups of the blend films shifted to lower wavenumber regions in comparison with the C=O stretching band of the chitosan film. In contrast, absorption bands of the –NH bending of the amide II groups of the blend films shifted to higher wavenumber regions compared to the –NH bending band of the chitosan film. This is attributed to the formation of hydrogen bonding interactions between the cellulose and chitosan, which enhanced the miscibility between them [4]. Therefore, it can be deduced that the preparation of cellulose/chitosan blend films in the [Bmim][OAc] ionic liquid has offered biopolymer blends with moderate thermal decomposition and good miscibility.

Besides that, the agarose/chitosan blend films have been prepared in the [Bmim][Cl] ionic liquid at different biopolymer weight ratios [30]. The thermal and chemical properties of the blend films were measured and analyzed by means of thermogravimetric analyzer and FTIR spectrometer, respectively. The thermal properties such as decomposition temperature of the agarose/chitosan blend film (20:80) moderately increased by up to 7.1% compared to the native agarose. This is due to the increase of chitosan content in the blend film, which changed its physical and molecular properties since the interactions between the agarose and chitosan established. Furthermore, the chemical properties such as absorption bands of the C=O stretching of the amide I groups of the blend films shifted to lower wavenumber regions as the content of agarose in the blend films is increased. The obtained results confirmed the formation of hydrogen bonding between the hydroxyl groups of agarose and the amino groups of chitosan [30]. Thus, it can be inferred that the preparation of agarose/chitosan blend films in the [Bmim][Cl] ionic liquid has provided biopolymer blends with moderate thermal decomposition and effective interactions.

The chitin/cellulose blend nanofibers have been prepared in the [Emim][OAc] ionic liquid at different biopolymer weight ratios [27]. The mechanical, thermal, and chemical properties of the blend nanofibers were characterized by using nanoindentation tester, thermogravimetric analyzer, and FTIR spectrometer, respectively. The mechanical properties such as indentation hardness and elastic modulus of the chitin/cellulose blend nanofiber (7:3) increased by up to 97% and 49%, respectively, compared to the regenerated chitin. This is caused by the addition of cellulose has influenced the strength/elasticity of the blend nanofiber, which disrupted the hydrogen bonding of CO···NH in the molecular chains of chitin. Nevertheless, the thermal properties such as decomposition temperature of the blend nanofibers decreased due to the new hydrogen bonding interactions between the chitin and cellulose. Moreover, the chemical properties such as absorption bands of the –NH– bending of the amide II groups of the blend nanofibers shifted to higher wavenumber regions as the cellulose content increased. This supported that the intermolecular hydrogen bonding of –HN···OH formed between the two biopolymers (–NH– of chitin and –OH of cellulose) [27]. Hence, it can be concluded that the preparation of chitin/cellulose blend nanofibers in the [Emim][OAc] ionic liquid has granted biopolymer blends with high strength/elasticity and effective interactions.

### 4.2. Physicochemical Properties of Polysaccharide/Polypeptide Blends

Table 8 shows the physicochemical properties of polysaccharide/polypeptide blends prepared in imidazolium-based ionic liquids. The cellulose/keratin blend filaments have been prepared in the [Emim][OAc] ionic liquid at different biopolymer weight ratios [8]. The mechanical and chemical properties of the blend filaments were measured and analyzed by means of tensile tester and FTIR spectrometer, respectively. The mechanical properties such as tenacity, tensile strength, Young’s modulus, stiffness, and elongation of the cellulose/keratin blend filament (90:10) increased by up to 81%, 14%, 34%, 21%, and 13%, respectively, compared to the regenerated cellulose. This indicated that the addition of keratin at low content had given a progressive effect on the strength properties of the blend filament. On the other hand, the chemical properties such as absorption bands of the C=O stretching of the amide I groups and C–N bending of the amide II groups of the blend filaments increase in the intensity. This is attributed to the increase of keratin content in the blend filaments [8]. Therefore, it can be deduced that the preparation of cellulose/keratin blend filaments in the [Emim][OAc] ionic liquid has given biopolymer blends with high tenacity and modulus properties.

The cellulose/keratin blend films have been prepared in the [Bmim][Cl] ionic liquid at different biopolymer weight ratios [6]. The mechanical, thermal, crystalline, and chemical properties of the blend films were characterized by using testing machine, thermogravimetric analyzer, wide-angle X-ray diffractometer, and FTIR spectrometer, respectively. The mechanical properties such as tensile strength and elongation at break of the cellulose/keratin blend film (70:30) moderately increased by up to 1.3% and 3.7%, respectively, compared to the regenerated cellulose. The increase is mostly related to the blend components in the film, compatibility, and interfacial adhesion between them. In addition, the thermal properties such as decomposition temperature of the blend film (70:30) increased to 306.8 °C in comparison with the regenerated cellulose. This is attributed to the compatibility between the cellulose and keratin, which improved the thermal stability of the blend film. Nonetheless, the crystalline properties such as crystallization peaks of the blend films decreased due to the decrease in crystallinity of the regenerated cellulose. This is also induced by the formation of hydrogen bonding with keratin. Furthermore, the chemical properties such as absorption bands of the C=O stretching of the amide I groups of the blend films shifted to lower wavenumber regions compared to the C=O stretching band of the regenerated cellulose. On the contrary, absorption bands of the –NH bending of the amide II groups of the blend films shifted to higher wavenumber regions in comparison with the –NH bending band of the regenerated keratin. This demonstrated that the intermolecular hydrogen bonding interactions have formed between the cellulose and keratin [6]. Hence, it can be inferred that the preparation of cellulose/keratin blend films in the [Bmim][Cl] ionic liquid has offered biopolymer blends with moderate elongation and thermal stability, as well as good compatibility.

Meanwhile, the cellulose/silk blend films have been prepared in the [Amim][Cl] ionic liquid at different biopolymer weight percentages [18]. The thermal, crystalline, and chemical properties of the blend films were characterized by using thermogravimetric analyzer, multi-angle X-ray diffractometer, and FTIR spectrometer, respectively. The thermal properties such as maximum decomposition temperature of the blend films decreased due to their homogeneous structures without immiscibility. Moreover, the crystalline properties such as semicrystalline nature of the blend films decreased because of the increase of silk content. On the other hand, the chemical properties such as absorption bands of the C=O stretching (amide I groups) and –NH bending (amide II groups) of the blend films shifted to higher wavenumber regions in comparison with the C=O stretching and –NH bending bands of the pure silk. This is attributed to the disruption of beta-sheets structural in the blend films by the intra- and intermolecular hydrogen bonding interactions between the cellulose and silk [18]. Thus, it can be concluded that the preparation of cellulose/silk blend films in the [Amim][Cl] ionic liquid has provided biopolymer blends with good miscibility.

On top of that, the cellulose/silk blend films have been prepared in the [Bmim][Cl] ionic liquid at different biopolymer weight ratios [14]. The mechanical, thermal, crystalline, and chemical properties of the blend films were characterized by using universal testing machine, thermogravimetric analyzer, X-ray diffractometer, and FTIR spectrometer, respectively. The mechanical properties such as tensile stress at break, tensile strain at break, and Young’s modulus of the blend films decreased because of the increase of silk content. This is also due to the poor mechanical properties of regenerated silk. Furthermore, the thermal properties such as decomposition temperature of the blend films decreased attributed to the addition of silk at high content, which increased the residual weight of the blend films. Additionally, the crystalline properties such as crystallinity pattern of the blend films decreased caused by the presence of silk molecular chains, which also implied miscibility between the cellulose and silk. On the other hand, the chemical properties such as absorption band of the C=O stretching of the amide I group of the blend film (75/25) shifted to a lower wavenumber region in comparison with the C=O stretching band of the regenerated silk. This is owing to the formation of strong hydrogen bonding interactions between the molecular chains of cellulose and silk [14]. Therefore, it can be deduced that the preparation of cellulose/silk blend films in the [Bmim][Cl] ionic liquid has granted biopolymer blends with good miscibility.

Besides that, the chitin/silk blend films have been prepared in the [Amim][Cl] ionic liquid at different biopolymer weight percentages [19]. The thermal, crystalline, and chemical properties of the blend films were characterized by using thermogravimetric analyzer, FSD-IR method, and FTIR spectrometer, respectively. The thermal properties such as maximum decomposition temperature of the blend films decreased because of the addition of silk, which acted as a thermal decomposition promoter for the blends. In addition, the crystalline properties such as crystallinity percentage of the chitin/silk blend film (20:80) increased by up to 88% in comparison with the blend film (80:20). This is attributed to the high content of silk could cause high crystallinity to the blend film. Moreover, the chemical properties such as absorption band of the –CN stretching of the amide III group of the blend film (80:20) increase in intensity. This displayed that the strong intermolecular hydrogen bonding interactions formed between the chitin and silk [19]. Hence, it can be inferred that the preparation of chitin/silk blend films in the [Amim][Cl] ionic liquid has given biopolymer blends with high crystallinity and good compatibility.

### 4.3. Physicochemical Properties of Polysaccharide/Bioplastic Blends

Table 9 exhibits the physicochemical properties of polysaccharide/bioplastic blends prepared in imidazolium-based ionic liquids. The cellulose/PCL blend films have been prepared in the [Bmim][Cl] ionic liquid at different polymer ratios [11]. The mechanical, thermal, crystalline, and chemical properties of the blend films were characterized by using dynamic mechanical analyzer, thermogravimetric analyzer, X-ray diffractometer, and FTIR spectrometer, respectively. The mechanical properties such as Young’s modulus, tensile strength, and elongation at break of the blend films decreased due to the increase of PCL content, which caused fragility to the blend films. Nevertheless, the thermal properties such as decomposition temperature of the cellulose/PCL blend film (40/60) increased by up to 30% compared to the neat cellulose. This is attributed to the presence of intramolecular interactions between the regenerated cellulose and PCL, which enhanced the thermal stability of the blend film. On the other hand, the crystalline properties such as crystallization peaks of the blend films decreased owing to the molecular chains of cellulose sterically inhibited the crystallization of PCL. However, the chemical properties such as absorption bands of the C=O stretching of the carbonyl groups of the blend films shifted to lower wavenumber regions in comparison with the C=O stretching band of the neat PCL. This confirmed that there are hydrogen bonding interactions between the hydroxyl groups of regenerated cellulose and carbonyl groups of PCL, which increased miscibility between them [11]. Therefore, it can be deduced that the preparation of cellulose/PCL blend films in the [Bmim][Cl] ionic liquid has offered polymer blends with high thermal decomposition and good miscibility.

The cellulose/PVA blend films have been prepared in the [Bmim][Cl] ionic liquid at different biopolymer weight ratios [12]. The mechanical, thermal, crystalline, and chemical properties of the blend films were characterized by using testing machine, differential scanning calorimeter, wide-angle X-ray diffractometer, and FTIR spectrometer, respectively. The mechanical properties such as Young’s modulus, tensile strength, and tensile strain of the cellulose/PVA blend film (70/30) increased by up to 69%, 23%, and 119%, respectively, compared to the regenerated cellulose. This implied that the addition of PVA significantly improved the mechanical properties of the blend film. Moreover, the thermal properties such as glass transition temperature of the blend film (60/40) increased by up to 17% in comparison with the regenerated PVA. This is due to the full miscibility of PVA with cellulose in the blend film. Nonetheless, the crystalline properties such as crystalline reflection peaks of the blend films decreased with increasing content of cellulose, which exhibited that the PVA crystallinity is decreased. On the other hand, the chemical properties such as absorption bands of the –OH stretching of the hydroxyl groups of the blend films shifted to higher wavenumber regions compared to the –OH stretching band of the regenerated cellulose. This revealed that the presence of intermolecular hydrogen bonding interactions between the hydroxyl groups of cellulose and PVA, which induced miscibility between them [12]. Hence, it can be concluded that the preparation of cellulose/PVA blend films in the [Bmim][Cl] ionic liquid has provided polymer blends with high modulus and glass transition temperature, as well as good miscibility.

Meanwhile, the cellulose/PHBV blend films have been prepared in the [Bmim][Cl] ionic liquid at different biopolymer weight ratios [13]. The mechanical, thermal, crystalline, and chemical properties of the blend films were characterized by using testing machine, differential scanning calorimeter, wide-angle X-ray diffractometer, and FTIR spectrometer, respectively. The mechanical properties such as Young’s modulus and tensile strength of the blend films decreased due to the addition of PHBV. This is also caused by the lower strength of regenerated PHBV than that of regenerated cellulose. Nevertheless, the thermal properties such as glass transition temperature of the cellulose/PHBV blend film (40/60) increased by up to 700% in comparison with the regenerated PHBV. This is attributed to the partial miscibility of PHBV with cellulose in their regenerated form. On the other hand, the crystalline properties such as crystalline reflection peaks of the blend films decreased owing to the low crystallization rate. This is also affected by the interactions between the cellulose and PHBV. Moreover, the chemical properties such as low-intensity absorption bands of the C=O stretching of the carbonyl groups of the blend films shifted to lower wavenumber regions compared to the C=O stretching band of the regenerated PHBV. This proved that there are partial hydrogen bonding interactions between the cellulose hydroxyl groups and PHBV carbonyl groups, which produced the blend films more compatible [13]. Thus, it can be inferred that the preparation of cellulose/PHBV blend films in the [Bmim][Cl] ionic liquid has granted polymer blends with high glass transition temperature and good compatibility.

Besides that, the chitin/PLA blend fibers have been prepared in the [Emim][OAc] ionic liquid at different biopolymer weight ratios [33]. The mechanical, thermal, and chemical properties of the blend fibers were characterized by using testing instrument, thermogravimetric analyzer, and FTIR spectrometer, respectively. The mechanical properties such as tensile strength, Young’s modulus, and tensile strain of the chitin/PLA blend fiber (1:0.3) increased by up to 58%, 40%, and 193%, respectively, compared to the neat chitin fiber. This displayed that the addition of PLA at low content has improved the strength properties of the blend fiber. Furthermore, the thermal properties such as onset decomposition temperature of the chitin/PLA blend fiber (1:0.1) increased by up to 19% in comparison with the chitin fiber. This is due to the homogenous blending between the chitin and PLA, which also depended on the PLA content. Additionally, the chemical properties such as absorption bands of the C=O stretching of the carbonyl groups of the blend fibers shifted to lower wavenumber regions compared to the C=O stretching band of the neat PLA. This confirmed that there are hydrogen bonding interactions between the carbonyl groups of PLA and the amide groups of chitin in the blend fibers, which enhanced their miscibility [33]. Therefore, it can be deduced that the preparation of chitin/PLA blend fibers in the [Emim][OAc] ionic liquid has given polymer blends with high strength and thermal decomposition, as well as good miscibility.

## 5. Conclusions

Biopolymer materials, imidazolium-based ionic liquids, types of biopolymer blends, and the physicochemical properties of biopolymer blends prepared in ionic liquids have been concisely reviewed in this paper. The vital physicochemical properties, for instance, mechanical, thermal, crystalline, and chemical of the blends, have also been determined in this concise review. Ionic liquids have commonly utilized for dissolution of biopolymers because they are non-volatile and recyclable. The recycling process of the ionic liquid can be conducted by removing a coagulation agent through evaporation, followed by freeze-drying. Ionic liquids used for the preparation of different types of biopolymer blends are mostly based on imidazolium cations combined with different counter anions. Additionally, [Amim][Cl], [Bmim][Cl], and [Emim][OAc] ionic liquids have been the three most significant ionic liquids for the biopolymer blends. [Amim][Cl] and [Emim][OAc] are frequently employed in the preparation of polysaccharide/polysaccharide blends and polysaccharide/polypeptide blends. Meanwhile, [Bmim][Cl] is typically used in the preparation of polysaccharide/bioplastic blends. The utilization of imidazolium-based ionic liquids could effectively improve the physicochemical properties of the biopolymer blends. Polysaccharide/polysaccharide blends possess high mechanical properties, moderate thermal decomposition, low crystalline properties, and good miscibility. On top of that, polysaccharide/polypeptide blends exhibit moderate mechanical properties, low thermal decomposition, low crystalline properties, and good compatibility. Besides that, polysaccharide/bioplastic blends have high mechanical and thermal properties, low crystalline properties, and good miscibility. The biopolymer blends prepared in imidazolium-based ionic liquids could form strong intermolecular hydrogen bonding interactions between the blend components. This concise review might be helpful not only for polymer blend researchers but also beneficial for commercializing biopolymer blends for diverse uses.

## Figures and Tables

**Table 1:** 1-Allyl-3-methylimidazolium-based ionic liquids with different counter anions utilized for dissolution of biopolymers.

Ionic Liquid	Abbreviation	Biopolymer	References
1-Allyl-3-methylimidazolium bromide	[Amim][Br]	Chitin	[17,26]
1-Allyl-3-methylimidazolium chloride	[Amim][Cl]	Cellulose	[7,18,19,20,22,25,29]
		Chitin	[19]
		Chitosan	[19,29]
		Keratin	[6]
		Lignin	[20]
		Silk	[18,19,22,25]
		Xylan	[20]

**Table 2:** 1-Butyl-3-methylimidazolium-based ionic liquids with different counter anions utilized for dissolution of biopolymers.

Ionic Liquid	Abbreviation	Biopolymer	References
1-Butyl-3-methylimidazolium chloride	[Bmim][Cl]	Cellulose	[2,6,7,9,13,14,16,17,21,22,24,26]
		Agarose	[30]
		Chitosan	[30]
		Collagen	[24]
		Keratin	[6,21]
		Silk	[2,14,22]
		Starch	[16]
		Zein	[16]
1-Butyl-3-methylimidazolium acetate	[Bmim][OAc]	Cellulose	[4,5]
		Chitosan	[4,5,35]
		Silk	[35]

**Table 3:** 1-Ethyl-3-methylimidazolium-based ionic liquids with different counter anions utilized for dissolution of biopolymers.

Ionic Liquid	Abbreviation	Biopolymer	References
1-Ethyl-3-methylimidazolium chloride	[Emim][Cl]	Cellulose	[15,22]
		Silk	[15,22]
1-Ethyl-3-methylimidazolium acetate	[Emim][OAc]	Cellulose	[5,7,8,15,22,23,27,28,43]
		Alginate	[31]
		Chitin	[27,31,33]
		Chitosan	[5,7]
		Keratin	[8]
		Silk	[15,22,28]
		Xylan	[43]
		Xyloglucan	[23]
1-Ethyl-3-methylimidazolium propionate	[Emim][OPr]	Cellulose	[10]
		Chitin	[10]

**Table 4:** Polysaccharides, ionic liquids, processing temperatures, and coagulation agents used for preparation of biopolymer blends.

Polysaccharide	Polysaccharide	Ionic Liquid	Processing Temperature (°C)	Coagulation Agent	References
Cellulose	Chitin	[Emim][OPr]	110	Water	[10]
Cellulose	Chitosan	[Emim][OAc]	110	Ethanol/water (1:1)	[7]
Cellulose	Chitosan	[Amim][Cl]	110	Water	[29]
Cellulose	Xylan	[Amim][Cl]	85	Water	[20]
Cellulose	Chitosan	[Bmim][OAc]	95	Methanol/water (1:1)	[4]
Agarose	Chitosan	[Bmim][Cl]	100	Methanol	[30]
Cellulose	Xyloglucan	[Emim][OAc]	100	Water or ethanol	[23]
Chitin	Cellulose	[Emim][OAc]	100	Water	[27]

**Table 5:** Polysaccharides, polypeptides, ionic liquids, processing temperatures, and coagulation agents used for preparation of biopolymer blends.

Polysaccharide	Polypeptide	Ionic Liquid	Processing Temperature (°C)	Coagulation Agent	References
Cellulose	Keratin	[Emim][OAc]	130	Ethanol	[8]
Cellulose	Keratin	[Bmim][Cl]	120	Water	[6]
Cellulose	Silk	[Amim][Cl]	85	Water	[18]
Chitin	Silk	[Amim][Cl]	103	Water	[19]
Chitosan	Silk	[Bmim][OAc]	95	Ethanol	[35]
Cellulose	Silk	[Bmim][Cl]	80	Methanol	[14]

**Table 6:** Polysaccharides, bioplastics, ionic liquids, processing temperatures, and coagulation agents used for preparation of biopolymer blends.

Polysaccharide	Bioplastic	Ionic Liquid	Processing Temperature (°C)	Coagulation Agent	References
Cellulose	PCL	[Bmim][Cl]	90	Water	[11]
Cellulose	PVA	[Bmim][Cl]	100	Ethanol	[12]
Cellulose	PHBV	[Bmim][Cl]	100	Water	[13]
Chitin	PLA	[Emim][OAc]	100	Water	[33]

**Table 7:** Physicochemical properties of polysaccharide/polysaccharide blends prepared in imidazolium-based ionic liquids.

Polymer Blend	Ionic Liquid	Physicochemical Properties *				References
		Mechanical	Thermal	Crystalline	Chemical	
Cellulose/chitin	[Emim][OPr]	↑	n/a	↑	n/a	[10]
Cellulose/chitosan	[Emim][OAc]	↓	↑	↓	↑	[7]
Cellulose/chitosan	[Amim][Cl]	↑	n/a	n/a	↑	[29]
Cellulose/chitosan	[Bmim][OAc]	n/a	↑	↓	↑	[4]
Agarose/chitosan	[Bmim][Cl]	n/a	↑	n/a	↑	[30]
Chitin/cellulose	[Emim][OAc]	↑	↓	n/a	↑	[27]

* The symbol ‘↑’ corresponds to an increase in the properties and ‘↓’ a decrease in the properties while ‘n/a’ describes not available.

**Table 8:** Physicochemical properties of polysaccharide/polypeptide blends prepared in imidazolium-based ionic liquids.

Polymer Blend	Ionic Liquid	Physicochemical Properties *				References
		Mechanical	Thermal	Crystalline	Chemical	
Cellulose/keratin	[Emim][OAc]	↑	n/a	n/a	↑	[8]
Cellulose/keratin	[Bmim][Cl]	↑	↑	↓	↑	[6]
Cellulose/silk	[Amim][Cl]	n/a	↓	↓	↑	[18]
Cellulose/silk	[Bmim][Cl]	↓	↓	↓	↑	[14]
Chitin/silk	[Amim][Cl]	n/a	↓	↑	↑	[19]

* The symbol ‘↑’ corresponds to an increase in the properties and ‘↓’ a decrease in the properties while ‘n/a’ describes not available.

**Table 9:** Physicochemical properties of polysaccharide/bioplastic blends prepared in imidazolium-based ionic liquids.

Polymer Blend	Ionic Liquid	Physicochemical Properties *				References
		Mechanical	Thermal	Crystalline	Chemical	
Cellulose/PCL	[Bmim][Cl]	↓	↑	↓	↑	[11]
Cellulose/PVA	[Bmim][Cl]	↑	↑	↓	↑	[12]
Cellulose/PHBV	[Bmim][Cl]	↓	↑	↓	↑	[13]
Chitin/PLA	[Emim][OAc]	↑	↑	n/a	↑	[33]

* The symbol ‘↑’ corresponds to an increase in the properties and ‘↓’ a decrease in the properties while ‘n/a’ describes not available.

## Data Availability

Not applicable.
